# Two mixed-ligand lanthanide–hydrazone complexes: [Pr(NCS)_3_(pbh)_2_]·H_2_O and [Nd(NCS)(NO_3_)(pbh)_2_(H_2_O)]NO_3_·2.33H_2_O [pbh is *N*′-(pyridin-2-ylmethylidene)benzo­hydrazide, C_13_H_11_N_3_O]

**DOI:** 10.1107/S2056989015024962

**Published:** 2016-01-16

**Authors:** Damianos G. Paschalidis, William T. A. Harrison

**Affiliations:** a Department of Chemistry, Aristotle University, 541 24 Thessaloniki, Greece; b Department of Chemistry, University of Aberdeen, Meston Walk, Aberdeen AB24 3UE, Scotland

**Keywords:** crystal structure, hydrazone, lanthanide, thio­cyanate, mixed ligands, hydrogen bonding.

## Abstract

The gel-mediated syntheses and crystal structures of [*N*′-(pyridin-2-ylmethylidene-κ*N*)benzohydrazide-κ^2^ *N*′,*O*]tris(thiocyanato-κ*N*)praseodymium(III) mono­hydrate, [Pr(NCS)_3_(C_13_H_11_N_3_O)_2_]·H_2_O, (I), and aqua(nitrato-κ^2^ *O*,*O*′)[*N*′-(pyri­din-2-ylmethylidene-κ*N*)benzohydrazide-κ^2^ *N*′,*O*](thiocyanato-κ*N*)neo­dym­ium(III) nitrate 2.33-hydrate, [Nd(NCS)(NO_3_)(C_13_H_11_N_3_O)_2_(H_2_O)]NO_3_·2.33H_2_O, (II), are reported. The Pr^3+^ ion in (I) is coordinated by two *N*,*N*,*O*-tridentate *N*′-(pyridin-2-ylmethylidene)benzohydrazide (pbh) ligands and three *N*-bonded thio­cyanate ions to generate an irregular PrN_7_O_2_ coordination polyhedron. The Nd^3+^ ion in (II) is coordinated by two *N*,*N*,*O*-tridentate pbh ligands, an *N*-bonded thio­cyanate ion, a bidentate nitrate ion and a water mol­ecule to generate a distorted NdN_5_O_5_ bicapped square anti­prism. The crystal structures of (I) and (II) feature numerous hydrogen bonds, which lead to the formation of three-dimensional networks in each case.

## Abstract 2

[Pr(C_13_H_11_N_3_O)_2_(NCS)_3_]·H_2_O contains an irregular PrN_7_O_2_ coordination polyhedron, whereas [Nd(C_13_H_11_N_3_O)_2_(NCS)(NO_3_)(H_2_O)](NO_3_)·2.33H_2_O contains a distorted NdN_5_O_5_ bicapped square anti­prism.

## Chemical context   

Hydrazones and their metal complexes show a wide range of properties and applications ranging from catalysts (Shibasaki & Yoshikawa, 2002 ▸), magnetization-transfer contrast agents (Zhang & Sherry, 2003 ▸) to light-emitting diodes (Kenyon, 2002 ▸). Our own studies in this area have focused on the syntheses and crystal structures of high-coordination-number lanthanide–hydrazone complexes including [Ce(NO_3_)_3_(pbh)_2_]C_3_H_6_O·2H_2_O (Christidis *et al.*, 1999 ▸), [Er(NO_3_)_2_(pbh)_2_]NO_3_·1.5H_2_O (Paschalidis *et al.*, 2000 ▸) and [Ce(pbh)_2_(NO_3_)(NCS)(H_2_O)]NO_3_·2.35H_2_O (Paschalidis & Gdaniec, 2004 ▸) [where pbh is pyridine-2-carboxaldehyde benzoyl­hydrazone].

As a continuation of these studies, we now describe the syntheses and crystal structures of the title mixed-ligand complexes [Pr(NCS)_3_(pbh)_2_]·H_2_O, (I),[Chem scheme1] and [Nd(NCS)(NO_3_)(pbh)_2_(H_2_O)](NO_3_)·2.33H_2_O, (II).

## Structural commentary   

**Figure 1:**
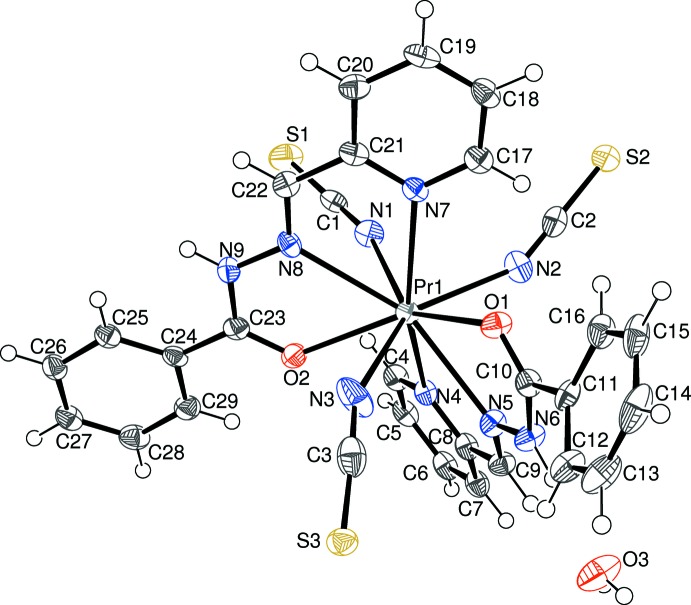
The mol­ecular structure of (I) showing 50% displacement ellipsoids and atom labelling.

**Figure 2:**
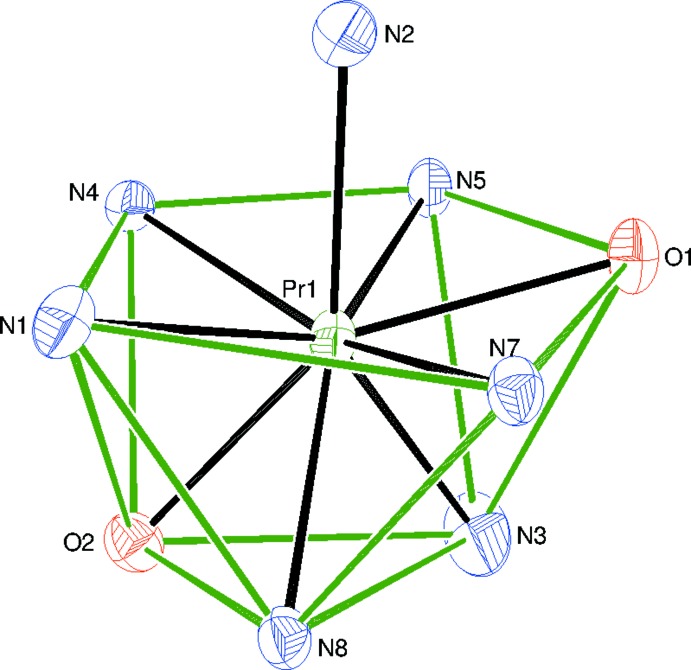
Detail of (I) showing the irregular PrO_2_N_7_ coordination polyhedron (contacts between the penta­gon and triangle of coordinated atoms shown as green lines). Displacement ellipsoids are shown at the 50% probability level.

Compound (I) is a new neutral mixed-ligand complex of Pr^3+^: selected geometrical data are given in Table 1 ▸. The praseodymium ion is coordinated by two *N*,*N*,*O*-tridentate (*via* the pyridine nitro­gen atom, the azomethine nitro­gen atom and the carbonyl oxygen atom) pbh ligands and three N-bonded thio­cyanate anions (Fig. 1 ▸), to yield a PrO_2_N_7_ coordination polyhedron for the metal ion (Fig. 2 ▸). Its geometry is irregular, but an approximate penta­gon of atoms N1/N4/N5/O1/N7 can be identified and a triangle of N3/N8/O2. The dihedral angle between these groups is 7.4 (2)° and the metal ion lies −1.898 (2) Å from the triangle and 0.5371 (13) Å from the mean plane of the penta­gon. Finally, atom N2 caps through the penta­gon at a distance of 1.947 (3) Å from its mean plane.
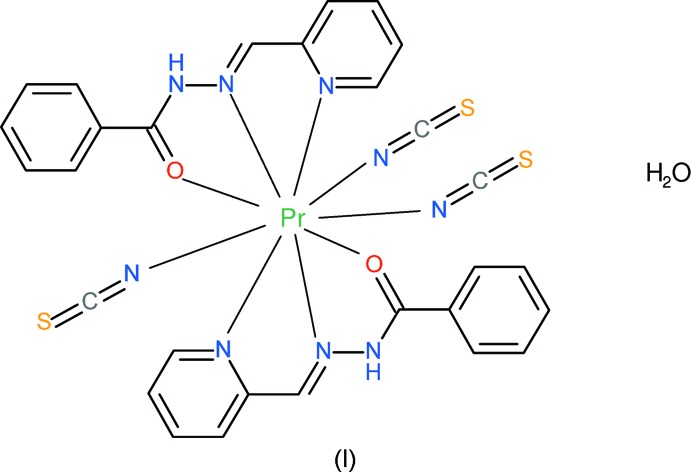
 
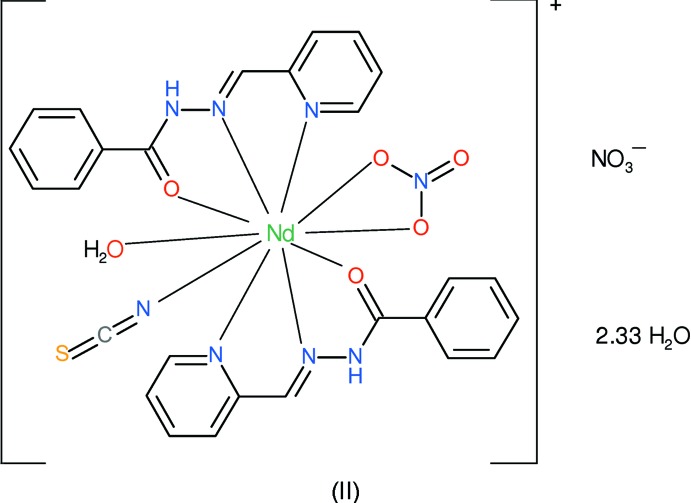


The first pbh ligand (containing C4) in (I) bonds to the metal ion from its atoms N4, N5 and O1, thus generating a pair of five-membered chelate rings. The first of these (N4/C8/C9/N5/Pr1) is almost planar (r.m.s. deviation = 0.011 Å) and the second (N5/N6/C10/O1/Pr1) can be described as a shallow envelope with O1 as the flap [displaced by 0.278 (4) Å from the mean plane through the other atoms with an r.m.s. deviation of 0.052 Å]. The dihedral angle between the N4/C4–C8 and C11–C16 aromatic rings of 49.44 (13)° indicates a substantial twisting to the ligand conformation: the major component to this occurs about the C10–C11 bond [N6—C10—C11—C12 = −37.1 (5)°]. For the second (C17) pbh ligand, atoms N7, N8 and O2 bond to the metal ion and the resulting chelate rings are both almost planar (for N7/C21/C22/N8/Pr1, r.m.s. deviation = 0.017 Å; for N8/N9/C23/O2/Pr1, r.m.s. deviation = 0.016 Å). The dihedral angle of 7.39 (9)° between the N7/C17–C21 and C24–C29 mean planes indicates that the second ligand is far less twisted than the first: the major component to this is reflected in the N9—C23—C24—C25 torsion angle of −11.3 (5)°. The dihedral angle between the near-planar parts of the pbh ligands (central chain plus pyridine ring) is 54.08 (6)°. The three thio­cyanate ligands show normal geometrical parameters (mean S=C bond length = 1.641 Å, mean C=N bond length = 1.169 Å, mean S=C=N bond angle = 179.0°): their Pr—N bond lengths are all shorter than the pbh Pr—N distances, which can be justified electrostatically if it is not a steric effect. The three Pr—N=C bond angles [159.0 (3), 150.7 (3) and 150.6 (3)°] are all substanti­ally less than 180°. A single water mol­ecule of crystallization completes the structure of (I).

**Figure 3:**
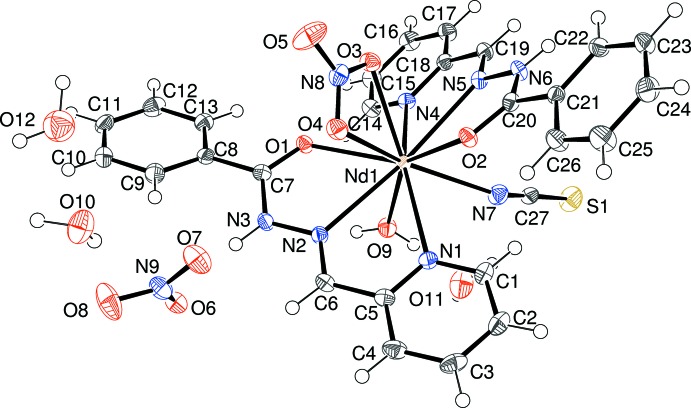
The mol­ecular structure of (II) showing 50% displacement ellipsoids and atom labelling.

**Figure 4:**
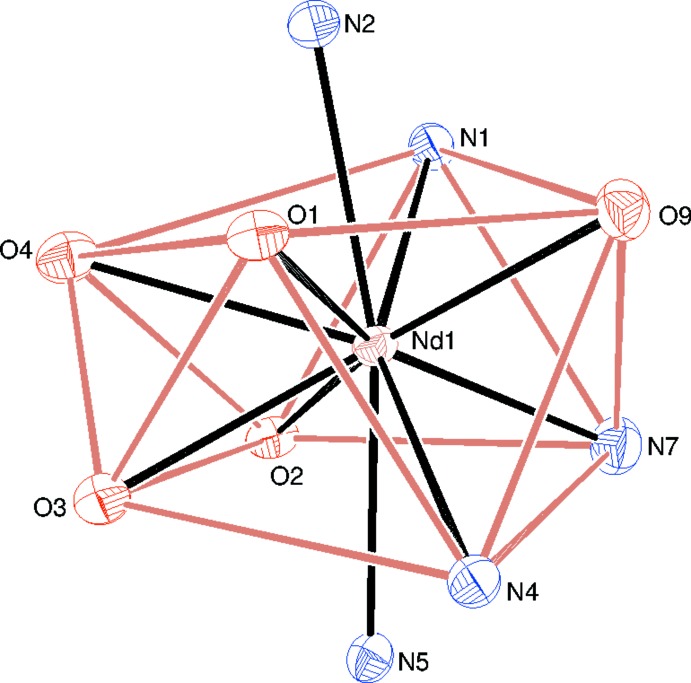
Detail of (II) showing the distorted bicapped square-anti­prismatic NdO_5_N_5_ coordination polyhedron (contacts between the atoms forming the square anti­prism indicated with tan lines). Displacement ellipsoids are shown at the 50% probability level.

Compound (II) is a new mixed-ligand cationic complex of Nd^3+^: selected geometrical data are given in Table 2 ▸. The neodymium ion is coordinated by two *N*,*N*,*O*-tridentate pbh ligands, an N-bonded thio­cyanate anion, a bidentate nitrate anion and a water mol­ecule (Fig. 3 ▸), to yield a 10-coordinate NdN_5_O_5_ coordination polyhedron. The coordination geometry about the Nd^3+^ ion (Fig. 4 ▸) at least approximates to a bicapped square anti­prism (Kepert, 1982 ▸) with the square faces defined by O1/O4/N1/O9 (r.m.s. deviation = 0.157 Å) and O2/O3/N4/N7 (r.m.s. deviation = 0.105 Å) and the capping atoms represented by N2 and N5 [N2—Nd1—N5 = 168.03 (6)°]. The dihedral angle between the nominal squares defined in the previous sentence is 8.11 (8)° and Nd1 is displaced from the afore-stated mean planes by −1.1431 (9) and 1.1762 (9) Å, respectively.

The first pbh ligand (containing C1) in (II) bonds to the metal ion from its atoms N1, N2 and O1. The two five-membered chelate rings that result are both close to planar (for N1/C5/C6/N2/Nd1, the r.m.s. deviation = 0.011 Å and for N2/N3/C7/O1/Nd1, the r.m.s. deviation = 0.019 Å). The dihedral angle between the N1/C1–C5 and C8–C13 aromatic rings is 21.71 (8)° and the metal ion is displaced from the pyridine ring by −0.204 (4) Å. For the second (C14) pbh ligand, atoms N4, N5 and O2 bond to the metal ion: one of the resulting chelate rings is close to planar (N4/C18/C19/N5/Nd1: r.m.s. deviation = 0.022 Å). The second (N5/N6/C20/O2/Nd1) is probably better described as a shallow envelope, with O2 displaced from the other atoms by −0.131 (3) Å. The dihedral angle of 9.52 (10)° between N4/C14–C18 and C21–C26 indicates that the second ligand is less twisted than the first. The metal ion is displaced by −0.045 (4) Å from the pyridine ring. The dihedral angle between the near-planar parts of the pbh ligands (central chain + pyridine ring) is 37.75 (3)°. The Nd—N—C bond angle of 149.40 (19)° is very similar to two of the corresponding angles in (I). The crystal structure of (II) is completed by a non-coordinating nitrate anion (also ensuring charge balance) and three water mol­ecules, one of which (O12) is partially occupied [refined occupancy = 0.328 (7)], although there are no close contacts that enforce this crystallographically.

## Supra­molecular features   

In the crystal of (I), the components are linked by N—H⋯Ow, N—H⋯S and Ow—H⋯S (w = water) hydrogen bonds (Table 3 ▸). The N—H⋯S bond generates [001] chains of complexes and the hydrogen bonds to and from the water mol­ecules generate a three-dimensional network. Aromatic π–π stacking between the N7-pyridine and C24-phenyl rings is suggested by the centroid–centroid separations of 3.524 (2) and 3.628 (2) Å between rings in nearby mol­ecules in the crystal and a short C—H⋯O contact (Table 3 ▸) also occurs.

In the crystal of (II), numerous hydrogen bonds occur (Table 4 ▸), to link the components into a three-dimensional network. Any aromatic π–π stacking must be very weak, as the minimum ring-centroid separation in the crystal is 3.9800 (13) Å.

## Database survey   

A search of the Cambridge Structural Database (Groom & Allen, 2014 ▸) for complexes incorporating pbh ligand(s) revealed 21 matches [two Group 1/2 metal ions (*N*,*O*-bidentate or *N*,*N*-tridentate), 16 transition metals (*N*,*N*-bidentate, *N*,*O*-bidentate or *N*,*N*,*O*-tridentate) and three lanthanides (all *N*,*N*,*O*-tridentate)]. The structure of the hydrated free ligand is also known (Richardson *et al.*, 1999 ▸). Based on this search, compound (I) appears to be a new structure type, whereas compound (II) is isostructural with its cerium analogue (refcode FEBDOG; Paschalidis & Gdaniec, 2004 ▸). Inter­estingly, both (II) and FEBDOG have almost the same occupancy factor for the partially occupied water mol­ecule.

## Synthesis and crystallization   

To prepare (I), gelled tetra­meth­oxy­silane (Arend & Connelly, 1982 ▸) was placed in the bend of a U-tube. A solution of 37.3 mg (0.1 mmol) PrCl_3_·6H_2_O and 22.8 mg (0.3 mmol) NH_4_SCN in 10 ml of methanol was placed in one arm of the tube and a solution of 45.0 mg (0.2 mmol) of pbh in 10 ml of methanol in the other. Green slabs of (I) were obtained after four months as the components slowly diffused through the gel. Analysis (%) calculated for C_29_H_24_N_9_O_3_PrS_3_: C, 44.44; H, 3.08; N, 16.08%. Found: C, 44.27; H, 3.01; N, 16.22%. IR (cm^−1^, KBr): 3445 *vw*, *b*, 2048 *vs* (NCS^−^ C≡N stretch), 1627 *s*, 1536 *s*, 1477 *m*, 1439 *m*, 1362 *m*, 1288 *m*, 1148 *m*, 1087 *w*, 1008 *w*, 919 *w*, 771 *w*, 710 *m*, 633 *w*.

To prepare (II), solutions of 43.8 mg (0.1 mmol) Nd(NO_3_)_3_·6H_2_O and 22.8 mg (0.3 mmol) NH_4_SCN in 10 ml of methanol and 45.0 mg (0.2 mmol) of pbh in 10 ml of methanol were placed in the arms of a U-tube filled with gelled tetra­meth­oxy­silane. Pale yellow slabs of (II) were obtained after four months. Analysis calculated for C_27_H_28.65_N_9_NdO_11.33_S: C, 38.75; H, 3.45; N, 15.06%. Found: C, 38.62; H, 3.41; N, 15.13%. IR (cm^−1^, KBr): 3447 *vw*, *b*, 2050 *vs* (NCS^−^ C≡N stretch), 1625 *s*, 1570 *s*, 1475 *m*, 1438 *m*, 1364 *m*, 1296 *m*, 1149 *m*, 1088 *w*, 1006 *w*, 920 *w*, 776 *w*, 700 *m*, 632 *w*.

## Refinement   

Crystal data, data collection and structure refinement details for (I) and (II) are summarized in Table 5 ▸. Atom O12 in (II) showed unrealistically large displacement parameters and its occupancy was refined to 0.327 (8). The O-bound H atoms were located in difference Fourier maps and refined as riding atoms in their as-found relative positions. The C- and N-bound H atoms were geometrically placed (C—H = 0.95–1.00 Å; N—H = 0.88 Å) and refined as riding atoms. The constraint *U*_iso_(H) = 1.2*U*_eq_(carrier) was applied in all cases.

## Supplementary Material

Crystal structure: contains datablock(s) I, II, global. DOI: 10.1107/S2056989015024962/su5267sup1.cif

Structure factors: contains datablock(s) I. DOI: 10.1107/S2056989015024962/su5267Isup2.hkl

Structure factors: contains datablock(s) II. DOI: 10.1107/S2056989015024962/su5267IIsup3.hkl

CCDC references: 1444956, 1444955

Additional supporting information:  crystallographic information; 3D view; checkCIF report

## Figures and Tables

**Table 1:** Selected bond lengths (Å) for (I)

Pr1—N2	2.485 (3)	Pr1—N8	2.646 (3)
Pr1—O2	2.498 (2)	Pr1—N5	2.666 (3)
Pr1—N1	2.517 (3)	Pr1—N4	2.674 (3)
Pr1—O1	2.529 (2)	Pr1—N7	2.679 (3)
Pr1—N3	2.550 (3)		

**Table 2:** Selected bond lengths (Å) for (II)

Nd1—O9	2.4459 (16)	Nd1—N5	2.6479 (19)
Nd1—O2	2.4796 (15)	Nd1—N2	2.6491 (18)
Nd1—O1	2.5063 (15)	Nd1—O4	2.6558 (17)
Nd1—N7	2.512 (2)	Nd1—N4	2.6985 (18)
Nd1—O3	2.5568 (17)	Nd1—N1	2.7051 (19)

**Table 3:** Hydrogen-bond geometry (Å, °) for (I)

*D*—H⋯*A*	*D*—H	H⋯*A*	*D*⋯*A*	*D*—H⋯*A*
N6—H6⋯O3	0.88	1.94	2.806 (4)	167
N9—H9⋯S3^i^	0.88	2.65	3.485 (3)	160
O3—H1⋯S2^ii^	0.84	2.46	3.278 (3)	164
O3—H2⋯S3^iii^	0.85	2.60	3.451 (3)	180
C26—H26⋯O1^iv^	0.95	2.53	3.450 (4)	162

Symmetry codes: (i) 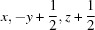; (ii) 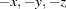; (iii) 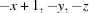; (iv) 
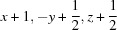
.

**Table 4:** Hydrogen-bond geometry (Å, °) for (II)

*D*—H⋯*A*	*D*—H	H⋯*A*	*D*⋯*A*	*D*—H⋯*A*
N3—H3⋯O6	0.88	1.98	2.847 (2)	168
N6—H6⋯O10^i^	0.88	1.92	2.754 (3)	159
O9—H1*W*⋯O11	0.90	1.86	2.760 (3)	174
O9—H2*W*⋯O6^ii^	0.90	1.93	2.816 (2)	168
O10—H3*W*⋯O5^iii^	0.99	2.07	3.055 (3)	171
O10—H4*W*⋯O8	0.94	1.95	2.824 (3)	154
O11—H5*W*⋯O5^iv^	0.94	1.94	2.859 (3)	166
O11—H6*W*⋯S1	0.93	2.58	3.460 (2)	159
O12—H7*W*⋯O7^iii^	0.95	1.95	2.902 (7)	180
O12—H8*W*⋯O5^iii^	0.90	2.25	3.072 (7)	151
O12—H8*W*⋯O4^iii^	0.90	2.14	2.957 (7)	149

Symmetry codes: (i) 
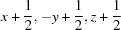
; (ii) 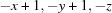; (iii) 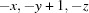; (iv) 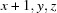.

**Table 5:** Experimental details

	(I)	(II)
Crystal data		
Chemical formula	[Pr(NCS)_3_(C_13_H_11_N_3_O)_2_]·H_2_O	[Nd(NCS)(NO_3_)(C_13_H_11_N_3_O)_2_(H_2_O)](NO_3_)·2.33H_2_O
*M*_r_	783.66	836.78
Crystal system, space group	Monoclinic, *P*2_1_/*c*	Monoclinic, *P*2_1_/*n*
Temperature (K)	120	120
*a*, *b*, *c* (Å)	9.6999 (4), 25.8275 (13), 13.5791 (7)	11.2796 (3), 17.3802 (3), 17.4298 (4)
β (°)	110.222 (2)	96.8035 (9)
*V* (Å^3^)	3192.2 (3)	3392.91 (13)
*Z*	4	4
Radiation type	Mo *K*α	Mo *K*α
μ (mm^−1^)	1.77	1.66
Crystal size (mm)	0.24 × 0.22 × 0.10	0.20 × 0.18 × 0.05

Data collection		
Diffractometer	Nonius KappaCCD	Nonius KappaCCD
Absorption correction	Multi-scan (*SADABS*; Bruker, 2003 ▸)	Multi-scan (*SADABS*; Bruker, 2003 ▸)
*T*_min_, *T*_max_	0.676, 0.843	0.732, 0.922
No. of measured, independent and observed [*I* > 2σ(*I*)] reflections	33021, 7291, 5239	41570, 7796, 6473
*R*_int_	0.059	0.038
(sin θ/λ)_max_ (Å^−1^)	0.650	0.651

Refinement		
*R*[*F* ^2^ > 2σ(*F* ^2^)], *wR*(*F* ^2^), *S*	0.037, 0.079, 1.04	0.026, 0.060, 1.03
No. of reflections	7291	7796
No. of parameters	406	447
H-atom treatment	H-atom parameters constrained	H-atom parameters constrained
Δρ_max_, Δρ_min_ (e Å^−3^)	1.21, −0.99	0.64, −0.50

Computer programs: *COLLECT* (Nonius, 1998 ▸), *HKL*, *SCALEPACK* and *DENZO* (Otwinowski & Minor, 1997 ▸) & *SORTAV* (Blessing, 1995 ▸), *SHELXS97* (Sheldrick, 2008 ▸), *SHELXL2014* (Sheldrick, 2015 ▸), *ORTEP-3 for Windows* (Farrugia, 2012 ▸) and *publCIF* (Westrip, 2010 ▸).

## supplementary crystallographic information

### (I) [N'-(Pyridin-2-ylmethylidene-κN)benzohydrazide-κ2N',O]tris(thiocyanato-κN)praseodymium(III) monohydrate Crystal data

**Table:**

[Pr(NCS)_3_(C_13_H_11_N_3_O)_2_]·H_2_O	*F*(000) = 1568
*M**_r_* = 783.66	*D*_x_ = 1.631 Mg m^−^^3^
Monoclinic, *P*2_1_/*c*	Mo *K*α radiation, λ = 0.71073 Å
*a* = 9.6999 (4) Å	Cell parameters from 6998 reflections
*b* = 25.8275 (13) Å	θ = 1.0–27.5°
*c* = 13.5791 (7) Å	µ = 1.77 mm^−^^1^
β = 110.222 (2)°	*T* = 120 K
*V* = 3192.2 (3) Å^3^	Slab, green
*Z* = 4	0.24 × 0.22 × 0.10 mm

### (I) [N'-(Pyridin-2-ylmethylidene-κN)benzohydrazide-κ2N',O]tris(thiocyanato-κN)praseodymium(III) monohydrate Data collection

**Table:**

Nonius KappaCCD diffractometer	5239 reflections with *I* > 2σ(*I*)
ω scans	*R*_int_ = 0.059
Absorption correction: multi-scan (*SADABS*; Bruker, 2003)	θ_max_ = 27.5°, θ_min_ = 1.8°
*T*_min_ = 0.676, *T*_max_ = 0.843	*h* = −12→12
33021 measured reflections	*k* = −33→33
7291 independent reflections	*l* = −17→17

### (I) [N'-(Pyridin-2-ylmethylidene-κN)benzohydrazide-κ2N',O]tris(thiocyanato-κN)praseodymium(III) monohydrate Refinement

**Table:**

Refinement on *F*^2^	Primary atom site location: structure-invariant direct methods
Least-squares matrix: full	Secondary atom site location: difference Fourier map
*R*[*F*^2^ > 2σ(*F*^2^)] = 0.037	Hydrogen site location: mixed
*wR*(*F*^2^) = 0.079	H-atom parameters constrained
*S* = 1.04	*w* = 1/[σ^2^(*F*_o_^2^) + (0.0324*P*)^2^ + 1.6778*P*] where *P* = (*F*_o_^2^ + 2*F*_c_^2^)/3
7291 reflections	(Δ/σ)_max_ < 0.001
406 parameters	Δρ_max_ = 1.21 e Å^−^^3^
0 restraints	Δρ_min_ = −0.99 e Å^−^^3^

### (I) [N'-(Pyridin-2-ylmethylidene-κN)benzohydrazide-κ2N',O]tris(thiocyanato-κN)praseodymium(III) monohydrate Special details

**Table:**

Geometry. All esds (except the esd in the dihedral angle between two l.s. planes) are estimated using the full covariance matrix. The cell esds are taken into account individually in the estimation of esds in distances, angles and torsion angles; correlations between esds in cell parameters are only used when they are defined by crystal symmetry. An approximate (isotropic) treatment of cell esds is used for estimating esds involving l.s. planes.

### (I) [N'-(Pyridin-2-ylmethylidene-κN)benzohydrazide-κ2N',O]tris(thiocyanato-κN)praseodymium(III) monohydrate Fractional atomic coordinates and isotropic or equivalent isotropic displacement parameters (Å^2^)

**Table:**

	*x*	*y*	*z*	*U*_iso_*/*U*_eq_	
Pr1	0.31829 (2)	0.12253 (2)	0.24529 (2)	0.02007 (7)	
N1	0.3645 (3)	0.13940 (12)	0.4366 (2)	0.0280 (7)	
C1	0.4060 (4)	0.16047 (14)	0.5174 (3)	0.0241 (8)	
S1	0.46404 (11)	0.19006 (4)	0.63040 (8)	0.0403 (3)	
N2	0.1460 (3)	0.06122 (12)	0.2837 (2)	0.0315 (7)	
C2	0.0465 (4)	0.05319 (14)	0.3096 (3)	0.0264 (8)	
S2	−0.09479 (11)	0.04185 (4)	0.34602 (8)	0.0351 (2)	
N3	0.4011 (4)	0.15427 (16)	0.0968 (3)	0.0477 (10)	
C3	0.4470 (4)	0.14588 (16)	0.0277 (3)	0.0398 (10)	
S3	0.51589 (11)	0.13680 (4)	−0.06694 (7)	0.0295 (2)	
C4	0.5853 (4)	0.04394 (15)	0.4307 (3)	0.0267 (8)	
H4	0.5825	0.0734	0.4719	0.032*	
C5	0.6773 (4)	0.00334 (15)	0.4786 (3)	0.0270 (8)	
H5	0.7354	0.0050	0.5510	0.032*	
C6	0.6833 (4)	−0.03941 (15)	0.4198 (3)	0.0283 (9)	
H6A	0.7461	−0.0676	0.4507	0.034*	
C7	0.5962 (4)	−0.04060 (14)	0.3149 (3)	0.0271 (8)	
H7	0.5977	−0.0697	0.2725	0.033*	
C8	0.5075 (4)	0.00113 (14)	0.2732 (3)	0.0232 (8)	
C9	0.4120 (4)	0.00156 (14)	0.1624 (3)	0.0251 (8)	
H9A	0.4141	−0.0261	0.1167	0.030*	
C10	0.1273 (4)	0.07720 (14)	0.0022 (3)	0.0244 (8)	
C11	0.0251 (4)	0.08091 (13)	−0.1085 (3)	0.0244 (8)	
C12	0.0722 (4)	0.07241 (14)	−0.1933 (3)	0.0327 (9)	
H12	0.1691	0.0606	−0.1823	0.039*	
C13	−0.0250 (5)	0.08149 (15)	−0.2936 (3)	0.0397 (10)	
H13	0.0060	0.0762	−0.3520	0.048*	
C14	−0.1662 (5)	0.09819 (15)	−0.3101 (3)	0.0421 (11)	
H14	−0.2311	0.1048	−0.3795	0.051*	
C15	−0.2136 (4)	0.10539 (15)	−0.2260 (3)	0.0381 (10)	
H15	−0.3117	0.1160	−0.2376	0.046*	
C16	−0.1178 (4)	0.09704 (14)	−0.1251 (3)	0.0309 (9)	
H16	−0.1496	0.1023	−0.0671	0.037*	
N4	0.5000 (3)	0.04383 (11)	0.3292 (2)	0.0224 (6)	
N5	0.3262 (3)	0.04014 (11)	0.1291 (2)	0.0244 (7)	
N6	0.2323 (3)	0.04032 (11)	0.0262 (2)	0.0264 (7)	
H6	0.2403	0.0180	−0.0205	0.032*	
O1	0.1171 (3)	0.10740 (9)	0.07046 (18)	0.0269 (6)	
C17	−0.0481 (4)	0.16983 (15)	0.1854 (3)	0.0257 (8)	
H17	−0.0657	0.1339	0.1718	0.031*	
C18	−0.1687 (4)	0.20226 (15)	0.1677 (3)	0.0279 (9)	
H18	−0.2656	0.1887	0.1432	0.034*	
C19	−0.1448 (4)	0.25442 (16)	0.1862 (3)	0.0283 (9)	
H19	−0.2250	0.2777	0.1729	0.034*	
C20	−0.0019 (4)	0.27227 (15)	0.2246 (3)	0.0269 (8)	
H20	0.0176	0.3080	0.2398	0.032*	
C21	0.1131 (4)	0.23752 (14)	0.2409 (2)	0.0227 (8)	
C22	0.2649 (4)	0.25490 (14)	0.2843 (3)	0.0239 (8)	
H22	0.2878	0.2899	0.3047	0.029*	
C23	0.6139 (4)	0.19972 (14)	0.3548 (2)	0.0224 (8)	
C24	0.7707 (4)	0.21404 (14)	0.4054 (3)	0.0218 (8)	
C25	0.8208 (4)	0.26462 (14)	0.4237 (3)	0.0240 (8)	
H25	0.7526	0.2924	0.4070	0.029*	
C26	0.9700 (4)	0.27478 (15)	0.4663 (3)	0.0256 (8)	
H26	1.0039	0.3095	0.4788	0.031*	
C27	1.0695 (4)	0.23443 (15)	0.4904 (3)	0.0279 (8)	
H27	1.1718	0.2414	0.5194	0.033*	
C28	1.0206 (4)	0.18410 (16)	0.4726 (3)	0.0319 (9)	
H28	1.0895	0.1565	0.4895	0.038*	
C29	0.8718 (4)	0.17344 (15)	0.4304 (3)	0.0276 (8)	
H29	0.8386	0.1386	0.4185	0.033*	
N7	0.0910 (3)	0.18629 (11)	0.2205 (2)	0.0213 (6)	
N8	0.3669 (3)	0.22167 (11)	0.2942 (2)	0.0219 (6)	
N9	0.5105 (3)	0.23700 (11)	0.3409 (2)	0.0231 (7)	
H9	0.5340	0.2692	0.3607	0.028*	
O2	0.5758 (2)	0.15496 (9)	0.32451 (18)	0.0253 (6)	
O3	0.2910 (3)	−0.03882 (11)	−0.0952 (2)	0.0510 (8)	
H1	0.2511	−0.0452	−0.1595	0.061*	
H2	0.3381	−0.0630	−0.0553	0.061*	

### (I) [N'-(Pyridin-2-ylmethylidene-κN)benzohydrazide-κ2N',O]tris(thiocyanato-κN)praseodymium(III) monohydrate Atomic displacement parameters (Å^2^)

**Table:**

	*U*^11^	*U*^22^	*U*^33^	*U*^12^	*U*^13^	*U*^23^
Pr1	0.01872 (10)	0.02491 (11)	0.01514 (9)	0.00104 (9)	0.00403 (7)	0.00080 (9)
N1	0.0310 (18)	0.0321 (18)	0.0210 (16)	0.0026 (14)	0.0091 (14)	0.0054 (14)
C1	0.0181 (18)	0.032 (2)	0.024 (2)	0.0050 (16)	0.0094 (16)	0.0070 (17)
S1	0.0298 (5)	0.0551 (7)	0.0285 (5)	0.0078 (5)	0.0007 (4)	−0.0129 (5)
N2	0.0279 (18)	0.035 (2)	0.0290 (17)	−0.0020 (15)	0.0068 (15)	0.0071 (15)
C2	0.027 (2)	0.022 (2)	0.0213 (18)	0.0017 (16)	−0.0028 (16)	0.0030 (15)
S2	0.0350 (6)	0.0357 (6)	0.0403 (6)	−0.0043 (5)	0.0201 (5)	−0.0058 (5)
N3	0.039 (2)	0.086 (3)	0.0230 (18)	−0.019 (2)	0.0159 (17)	0.0056 (18)
C3	0.034 (2)	0.032 (2)	0.045 (3)	−0.0041 (19)	0.003 (2)	0.008 (2)
S3	0.0361 (5)	0.0276 (5)	0.0291 (5)	0.0015 (4)	0.0168 (4)	0.0014 (4)
C4	0.025 (2)	0.034 (2)	0.0201 (18)	−0.0026 (17)	0.0062 (16)	0.0016 (16)
C5	0.0196 (19)	0.039 (2)	0.0193 (18)	−0.0004 (17)	0.0029 (15)	0.0072 (16)
C6	0.0195 (19)	0.031 (2)	0.031 (2)	0.0015 (16)	0.0047 (16)	0.0109 (17)
C7	0.0206 (18)	0.028 (2)	0.030 (2)	0.0002 (16)	0.0057 (16)	0.0039 (16)
C8	0.0165 (18)	0.025 (2)	0.0241 (18)	0.0015 (15)	0.0017 (15)	0.0059 (16)
C9	0.0239 (19)	0.027 (2)	0.0227 (18)	0.0046 (17)	0.0062 (16)	−0.0008 (16)
C10	0.0227 (19)	0.027 (2)	0.0209 (18)	−0.0005 (16)	0.0050 (15)	0.0017 (16)
C11	0.029 (2)	0.0182 (19)	0.0191 (18)	−0.0003 (16)	0.0001 (16)	−0.0025 (15)
C12	0.039 (2)	0.028 (2)	0.026 (2)	0.0067 (18)	0.0046 (18)	−0.0018 (17)
C13	0.062 (3)	0.030 (2)	0.020 (2)	0.006 (2)	0.005 (2)	−0.0011 (17)
C14	0.052 (3)	0.026 (2)	0.027 (2)	−0.001 (2)	−0.014 (2)	−0.0004 (18)
C15	0.028 (2)	0.027 (2)	0.041 (2)	−0.0034 (17)	−0.0104 (19)	−0.0016 (18)
C16	0.025 (2)	0.026 (2)	0.033 (2)	0.0006 (17)	0.0004 (17)	−0.0002 (18)
N4	0.0200 (15)	0.0239 (17)	0.0216 (15)	−0.0010 (13)	0.0048 (13)	0.0035 (13)
N5	0.0229 (16)	0.0271 (17)	0.0185 (15)	0.0019 (14)	0.0013 (13)	0.0009 (13)
N6	0.0256 (16)	0.0316 (18)	0.0142 (14)	0.0077 (14)	−0.0028 (13)	−0.0033 (13)
O1	0.0263 (14)	0.0326 (15)	0.0182 (12)	0.0063 (11)	0.0030 (11)	−0.0023 (11)
C17	0.026 (2)	0.033 (2)	0.0187 (18)	0.0000 (17)	0.0079 (16)	0.0017 (16)
C18	0.0202 (19)	0.043 (3)	0.0195 (18)	−0.0029 (18)	0.0053 (15)	−0.0014 (17)
C19	0.0209 (19)	0.046 (3)	0.0193 (18)	0.0108 (18)	0.0079 (16)	0.0027 (17)
C20	0.028 (2)	0.031 (2)	0.0229 (18)	0.0050 (17)	0.0100 (16)	−0.0006 (16)
C21	0.0214 (18)	0.031 (2)	0.0150 (17)	0.0049 (16)	0.0057 (15)	0.0028 (15)
C22	0.025 (2)	0.025 (2)	0.0202 (18)	0.0016 (16)	0.0062 (16)	0.0010 (15)
C23	0.027 (2)	0.028 (2)	0.0145 (16)	−0.0005 (17)	0.0105 (15)	0.0006 (15)
C24	0.0206 (18)	0.031 (2)	0.0167 (17)	−0.0008 (16)	0.0096 (15)	−0.0020 (15)
C25	0.0228 (19)	0.029 (2)	0.0203 (18)	0.0006 (16)	0.0080 (15)	0.0010 (15)
C26	0.026 (2)	0.031 (2)	0.0213 (18)	−0.0048 (17)	0.0093 (16)	−0.0019 (16)
C27	0.0202 (19)	0.040 (2)	0.0215 (18)	−0.0010 (17)	0.0052 (16)	−0.0009 (17)
C28	0.024 (2)	0.040 (3)	0.031 (2)	0.0070 (18)	0.0078 (17)	−0.0007 (18)
C29	0.026 (2)	0.031 (2)	0.0257 (19)	−0.0005 (17)	0.0086 (17)	−0.0044 (16)
N7	0.0194 (15)	0.0293 (18)	0.0151 (14)	−0.0004 (13)	0.0061 (12)	0.0004 (12)
N8	0.0170 (15)	0.0282 (18)	0.0186 (15)	−0.0024 (13)	0.0039 (12)	0.0021 (13)
N9	0.0215 (16)	0.0224 (17)	0.0242 (16)	−0.0026 (13)	0.0065 (13)	−0.0009 (13)
O2	0.0240 (13)	0.0246 (14)	0.0266 (13)	0.0003 (11)	0.0078 (11)	−0.0034 (11)
O3	0.061 (2)	0.0457 (19)	0.0356 (17)	0.0210 (16)	0.0030 (15)	−0.0086 (14)

### (I) [N'-(Pyridin-2-ylmethylidene-κN)benzohydrazide-κ2N',O]tris(thiocyanato-κN)praseodymium(III) monohydrate Geometric parameters (Å, º)

**Table:**

Pr1—N2	2.485 (3)	C14—H14	0.9500
Pr1—O2	2.498 (2)	C15—C16	1.381 (5)
Pr1—N1	2.517 (3)	C15—H15	0.9500
Pr1—O1	2.529 (2)	C16—H16	0.9500
Pr1—N3	2.550 (3)	N5—N6	1.380 (4)
Pr1—N8	2.646 (3)	N6—H6	0.8800
Pr1—N5	2.666 (3)	C17—N7	1.335 (4)
Pr1—N4	2.674 (3)	C17—C18	1.390 (5)
Pr1—N7	2.679 (3)	C17—H17	0.9500
N1—C1	1.165 (4)	C18—C19	1.375 (5)
C1—S1	1.630 (4)	C18—H18	0.9500
N2—C2	1.154 (4)	C19—C20	1.381 (5)
C2—S2	1.636 (4)	C19—H19	0.9500
N3—C3	1.189 (5)	C20—C21	1.388 (5)
C3—S3	1.658 (5)	C20—H20	0.9500
C4—N4	1.340 (4)	C21—N7	1.353 (4)
C4—C5	1.385 (5)	C21—C22	1.455 (5)
C4—H4	0.9500	C22—N8	1.281 (4)
C5—C6	1.376 (5)	C22—H22	0.9500
C5—H5	0.9500	C23—O2	1.240 (4)
C6—C7	1.382 (5)	C23—N9	1.356 (4)
C6—H6A	0.9500	C23—C24	1.483 (5)
C7—C8	1.373 (5)	C24—C25	1.386 (5)
C7—H7	0.9500	C24—C29	1.395 (5)
C8—N4	1.355 (4)	C25—C26	1.386 (5)
C8—C9	1.470 (5)	C25—H25	0.9500
C9—N5	1.276 (4)	C26—C27	1.380 (5)
C9—H9A	0.9500	C26—H26	0.9500
C10—O1	1.241 (4)	C27—C28	1.376 (5)
C10—N6	1.350 (4)	C27—H27	0.9500
C10—C11	1.490 (5)	C28—C29	1.384 (5)
C11—C16	1.389 (5)	C28—H28	0.9500
C11—C12	1.395 (5)	C29—H29	0.9500
C12—C13	1.383 (5)	N8—N9	1.374 (4)
C12—H12	0.9500	N9—H9	0.8800
C13—C14	1.378 (6)	O3—H1	0.8395
C13—H13	0.9500	O3—H2	0.8498
C14—C15	1.384 (6)		
			
N2—Pr1—O2	140.19 (9)	C12—C13—H13	119.5
N2—Pr1—N1	77.66 (10)	C13—C14—C15	120.2 (4)
O2—Pr1—N1	72.71 (9)	C13—C14—H14	119.9
N2—Pr1—O1	75.04 (9)	C15—C14—H14	119.9
O2—Pr1—O1	142.03 (8)	C16—C15—C14	119.7 (4)
N1—Pr1—O1	143.14 (9)	C16—C15—H15	120.1
N2—Pr1—N3	142.12 (11)	C14—C15—H15	120.1
O2—Pr1—N3	72.02 (9)	C15—C16—C11	120.0 (4)
N1—Pr1—N3	140.19 (11)	C15—C16—H16	120.0
O1—Pr1—N3	70.07 (9)	C11—C16—H16	120.0
N2—Pr1—N8	129.67 (9)	C4—N4—C8	116.4 (3)
O2—Pr1—N8	60.17 (8)	C4—N4—Pr1	121.2 (2)
N1—Pr1—N8	67.72 (9)	C8—N4—Pr1	122.3 (2)
O1—Pr1—N8	113.44 (8)	C9—N5—N6	118.8 (3)
N3—Pr1—N8	79.03 (11)	C9—N5—Pr1	124.2 (2)
N2—Pr1—N5	76.96 (10)	N6—N5—Pr1	116.9 (2)
O2—Pr1—N5	107.07 (8)	C10—N6—N5	115.2 (3)
N1—Pr1—N5	135.86 (9)	C10—N6—H6	122.4
O1—Pr1—N5	59.45 (8)	N5—N6—H6	122.4
N3—Pr1—N5	72.99 (11)	C10—O1—Pr1	124.3 (2)
N8—Pr1—N5	151.86 (8)	N7—C17—C18	123.7 (4)
N2—Pr1—N4	79.53 (9)	N7—C17—H17	118.1
O2—Pr1—N4	69.97 (8)	C18—C17—H17	118.1
N1—Pr1—N4	80.55 (9)	C19—C18—C17	118.7 (3)
O1—Pr1—N4	117.63 (8)	C19—C18—H18	120.6
N3—Pr1—N4	103.88 (11)	C17—C18—H18	120.6
N8—Pr1—N4	126.48 (8)	C18—C19—C20	118.7 (3)
N5—Pr1—N4	59.72 (9)	C18—C19—H19	120.7
N2—Pr1—N7	80.44 (10)	C20—C19—H19	120.7
O2—Pr1—N7	120.34 (8)	C19—C20—C21	119.4 (4)
N1—Pr1—N7	82.42 (9)	C19—C20—H20	120.3
O1—Pr1—N7	69.06 (8)	C21—C20—H20	120.3
N3—Pr1—N7	100.01 (10)	N7—C21—C20	122.5 (3)
N8—Pr1—N7	60.28 (8)	N7—C21—C22	116.8 (3)
N5—Pr1—N7	127.39 (8)	C20—C21—C22	120.6 (3)
N4—Pr1—N7	156.03 (8)	N8—C22—C21	118.2 (3)
C1—N1—Pr1	159.0 (3)	N8—C22—H22	120.9
N1—C1—S1	179.9 (4)	C21—C22—H22	120.9
C2—N2—Pr1	150.7 (3)	O2—C23—N9	119.7 (3)
N2—C2—S2	179.9 (4)	O2—C23—C24	121.8 (3)
C3—N3—Pr1	150.6 (3)	N9—C23—C24	118.6 (3)
N3—C3—S3	177.2 (4)	C25—C24—C29	119.4 (3)
N4—C4—C5	123.4 (4)	C25—C24—C23	123.9 (3)
N4—C4—H4	118.3	C29—C24—C23	116.6 (3)
C5—C4—H4	118.3	C26—C25—C24	120.3 (3)
C6—C5—C4	119.0 (3)	C26—C25—H25	119.9
C6—C5—H5	120.5	C24—C25—H25	119.9
C4—C5—H5	120.5	C27—C26—C25	120.0 (3)
C5—C6—C7	118.9 (3)	C27—C26—H26	120.0
C5—C6—H6A	120.6	C25—C26—H26	120.0
C7—C6—H6A	120.6	C28—C27—C26	120.1 (3)
C8—C7—C6	118.7 (4)	C28—C27—H27	120.0
C8—C7—H7	120.7	C26—C27—H27	120.0
C6—C7—H7	120.7	C27—C28—C29	120.5 (4)
N4—C8—C7	123.7 (3)	C27—C28—H28	119.8
N4—C8—C9	115.5 (3)	C29—C28—H28	119.8
C7—C8—C9	120.8 (3)	C28—C29—C24	119.7 (4)
N5—C9—C8	118.1 (3)	C28—C29—H29	120.1
N5—C9—H9A	121.0	C24—C29—H29	120.1
C8—C9—H9A	121.0	C17—N7—C21	117.0 (3)
O1—C10—N6	120.8 (3)	C17—N7—Pr1	122.4 (2)
O1—C10—C11	121.1 (3)	C21—N7—Pr1	120.6 (2)
N6—C10—C11	118.2 (3)	C22—N8—N9	118.6 (3)
C16—C11—C12	120.4 (3)	C22—N8—Pr1	123.9 (2)
C16—C11—C10	117.5 (3)	N9—N8—Pr1	117.3 (2)
C12—C11—C10	121.9 (3)	C23—N9—N8	116.3 (3)
C13—C12—C11	118.7 (4)	C23—N9—H9	121.9
C13—C12—H12	120.6	N8—N9—H9	121.9
C11—C12—H12	120.6	C23—O2—Pr1	126.5 (2)
C14—C13—C12	121.0 (4)	H1—O3—H2	118.2
C14—C13—H13	119.5		
			
N4—C4—C5—C6	0.4 (5)	N7—C17—C18—C19	0.6 (5)
C4—C5—C6—C7	−0.5 (5)	C17—C18—C19—C20	−1.8 (5)
C5—C6—C7—C8	0.3 (5)	C18—C19—C20—C21	1.6 (5)
C6—C7—C8—N4	0.0 (5)	C19—C20—C21—N7	−0.1 (5)
C6—C7—C8—C9	−179.5 (3)	C19—C20—C21—C22	−178.1 (3)
N4—C8—C9—N5	−3.2 (5)	N7—C21—C22—N8	4.3 (5)
C7—C8—C9—N5	176.4 (3)	C20—C21—C22—N8	−177.5 (3)
O1—C10—C11—C16	−32.9 (5)	O2—C23—C24—C25	168.1 (3)
N6—C10—C11—C16	147.6 (3)	N9—C23—C24—C25	−11.3 (5)
O1—C10—C11—C12	142.3 (4)	O2—C23—C24—C29	−8.7 (5)
N6—C10—C11—C12	−37.1 (5)	N9—C23—C24—C29	171.9 (3)
C16—C11—C12—C13	1.5 (6)	C29—C24—C25—C26	0.2 (5)
C10—C11—C12—C13	−173.7 (4)	C23—C24—C25—C26	−176.5 (3)
C11—C12—C13—C14	−0.6 (6)	C24—C25—C26—C27	0.1 (5)
C12—C13—C14—C15	−1.0 (6)	C25—C26—C27—C28	−0.2 (5)
C13—C14—C15—C16	1.6 (6)	C26—C27—C28—C29	0.0 (5)
C14—C15—C16—C11	−0.7 (6)	C27—C28—C29—C24	0.2 (5)
C12—C11—C16—C15	−0.8 (6)	C25—C24—C29—C28	−0.3 (5)
C10—C11—C16—C15	174.5 (3)	C23—C24—C29—C28	176.6 (3)
C5—C4—N4—C8	−0.1 (5)	C18—C17—N7—C21	1.0 (5)
C5—C4—N4—Pr1	177.7 (2)	C18—C17—N7—Pr1	−177.3 (2)
C7—C8—N4—C4	−0.1 (5)	C20—C21—N7—C17	−1.2 (5)
C9—C8—N4—C4	179.4 (3)	C22—C21—N7—C17	176.9 (3)
C7—C8—N4—Pr1	−177.9 (2)	C20—C21—N7—Pr1	177.1 (2)
C9—C8—N4—Pr1	1.7 (4)	C22—C21—N7—Pr1	−4.8 (4)
C8—C9—N5—N6	−177.9 (3)	C21—C22—N8—N9	−176.1 (3)
C8—C9—N5—Pr1	3.3 (4)	C21—C22—N8—Pr1	−1.8 (4)
O1—C10—N6—N5	−1.5 (5)	O2—C23—N9—N8	0.9 (4)
C11—C10—N6—N5	177.9 (3)	C24—C23—N9—N8	−179.7 (3)
C9—N5—N6—C10	168.3 (3)	C22—N8—N9—C23	176.5 (3)
Pr1—N5—N6—C10	−12.7 (4)	Pr1—N8—N9—C23	1.7 (3)
N6—C10—O1—Pr1	17.0 (5)	N9—C23—O2—Pr1	−3.5 (4)
C11—C10—O1—Pr1	−162.4 (2)	C24—C23—O2—Pr1	177.1 (2)

### (I) [N'-(Pyridin-2-ylmethylidene-κN)benzohydrazide-κ2N',O]tris(thiocyanato-κN)praseodymium(III) monohydrate Hydrogen-bond geometry (Å, º)

**Table:**

*D*—H···*A*	*D*—H	H···*A*	*D*···*A*	*D*—H···*A*
N6—H6···O3	0.88	1.94	2.806 (4)	167
N9—H9···S3^i^	0.88	2.65	3.485 (3)	160
O3—H1···S2^ii^	0.84	2.46	3.278 (3)	164
O3—H2···S3^iii^	0.85	2.60	3.451 (3)	180
C26—H26···O1^iv^	0.95	2.53	3.450 (4)	162

Symmetry codes: (i) *x*, −*y*+1/2, *z*+1/2; (ii) −*x*, −*y*, −*z*; (iii) −*x*+1, −*y*, −*z*; (iv) *x*+1, −*y*+1/2, *z*+1/2.

### (II) Aqua(nitrato-κ2O,O')[N'-(pyridin-2-ylmethylidene-κN)benzohydrazide-κ2N',O](thiocyanato-κN)neodymium(III) nitrate 2.33-hydrate Crystal data

**Table:**

[Nd(NCS)(NO_3_)(C_13_H_11_N_3_O)_2_(H_2_O)]NO_3_·2.33H_2_O	*F*(000) = 1681
*M**_r_* = 836.78	*D*_x_ = 1.638 Mg m^−^^3^
Monoclinic, *P*2_1_/*n*	Mo *K*α radiation, λ = 0.70173 Å
*a* = 11.2796 (3) Å	Cell parameters from 7981 reflections
*b* = 17.3802 (3) Å	θ = 2.9–27.5°
*c* = 17.4298 (4) Å	µ = 1.66 mm^−^^1^
β = 96.8035 (9)°	*T* = 120 K
*V* = 3392.91 (13) Å^3^	Slab, light yellow-brown
*Z* = 4	0.20 × 0.18 × 0.05 mm

### (II) Aqua(nitrato-κ2O,O')[N'-(pyridin-2-ylmethylidene-κN)benzohydrazide-κ2N',O](thiocyanato-κN)neodymium(III) nitrate 2.33-hydrate Data collection

**Table:**

Nonius KappaCCD diffractometer	6473 reflections with *I* > 2σ(*I*)
ω scans	*R*_int_ = 0.038
Absorption correction: multi-scan (*SADABS*; Bruker, 2003)	θ_max_ = 27.2°, θ_min_ = 2.9°
*T*_min_ = 0.732, *T*_max_ = 0.922	*h* = −14→14
41570 measured reflections	*k* = −22→22
7796 independent reflections	*l* = −22→22

### (II) Aqua(nitrato-κ2O,O')[N'-(pyridin-2-ylmethylidene-κN)benzohydrazide-κ2N',O](thiocyanato-κN)neodymium(III) nitrate 2.33-hydrate Refinement

**Table:**

Refinement on *F*^2^	0 restraints
Least-squares matrix: full	Hydrogen site location: mixed
*R*[*F*^2^ > 2σ(*F*^2^)] = 0.026	H-atom parameters constrained
*wR*(*F*^2^) = 0.060	*w* = 1/[σ^2^(*F*_o_^2^) + (0.0194*P*)^2^ + 4.1799*P*] where *P* = (*F*_o_^2^ + 2*F*_c_^2^)/3
*S* = 1.03	(Δ/σ)_max_ = 0.001
7796 reflections	Δρ_max_ = 0.64 e Å^−^^3^
447 parameters	Δρ_min_ = −0.50 e Å^−^^3^

### (II) Aqua(nitrato-κ2O,O')[N'-(pyridin-2-ylmethylidene-κN)benzohydrazide-κ2N',O](thiocyanato-κN)neodymium(III) nitrate 2.33-hydrate Special details

**Table:**

Geometry. All esds (except the esd in the dihedral angle between two l.s. planes) are estimated using the full covariance matrix. The cell esds are taken into account individually in the estimation of esds in distances, angles and torsion angles; correlations between esds in cell parameters are only used when they are defined by crystal symmetry. An approximate (isotropic) treatment of cell esds is used for estimating esds involving l.s. planes.

### (II) Aqua(nitrato-κ2O,O')[N'-(pyridin-2-ylmethylidene-κN)benzohydrazide-κ2N',O](thiocyanato-κN)neodymium(III) nitrate 2.33-hydrate Fractional atomic coordinates and isotropic or equivalent isotropic displacement parameters (Å^2^)

**Table:**

	*x*	*y*	*z*	*U*_iso_*/*U*_eq_	Occ. (<1)
Nd1	0.44943 (2)	0.25257 (2)	0.12087 (2)	0.01344 (4)	
C1	0.4747 (2)	0.12840 (14)	−0.04159 (15)	0.0224 (5)	
H1	0.4930	0.0937	0.0002	0.027*	
C2	0.4687 (2)	0.10014 (15)	−0.11614 (15)	0.0262 (5)	
H2	0.4811	0.0470	−0.1250	0.031*	
C3	0.4443 (2)	0.14999 (15)	−0.17748 (15)	0.0280 (6)	
H3A	0.4406	0.1320	−0.2292	0.034*	
C4	0.4255 (2)	0.22674 (15)	−0.16222 (14)	0.0256 (5)	
H4	0.4086	0.2624	−0.2033	0.031*	
C5	0.4314 (2)	0.25089 (13)	−0.08627 (13)	0.0180 (5)	
C6	0.4103 (2)	0.33129 (13)	−0.06820 (13)	0.0187 (5)	
H6A	0.3952	0.3687	−0.1079	0.022*	
C7	0.39161 (19)	0.44243 (13)	0.09589 (13)	0.0165 (5)	
C8	0.3629 (2)	0.52246 (13)	0.11605 (13)	0.0187 (5)	
C9	0.2952 (3)	0.57111 (15)	0.06426 (15)	0.0291 (6)	
H9	0.2653	0.5529	0.0143	0.035*	
C10	0.2715 (3)	0.64582 (16)	0.08559 (16)	0.0338 (7)	
H10	0.2267	0.6790	0.0498	0.041*	
C11	0.3125 (2)	0.67218 (15)	0.15852 (16)	0.0284 (6)	
H11	0.2961	0.7234	0.1730	0.034*	
C12	0.3775 (2)	0.62378 (15)	0.21046 (16)	0.0271 (6)	
H12	0.4049	0.6418	0.2609	0.032*	
C13	0.4033 (2)	0.54927 (14)	0.18963 (14)	0.0208 (5)	
H13	0.4486	0.5165	0.2256	0.025*	
N1	0.45601 (17)	0.20273 (11)	−0.02559 (11)	0.0180 (4)	
N2	0.41293 (17)	0.34981 (11)	0.00340 (11)	0.0165 (4)	
N3	0.39226 (17)	0.42549 (11)	0.02046 (11)	0.0182 (4)	
H3	0.3802	0.4608	−0.0158	0.022*	
O1	0.41579 (14)	0.39231 (9)	0.14645 (9)	0.0177 (3)	
C14	0.6017 (2)	0.35445 (14)	0.28346 (13)	0.0201 (5)	
H14	0.6054	0.3912	0.2434	0.024*	
C15	0.6504 (2)	0.37410 (14)	0.35775 (14)	0.0226 (5)	
H15	0.6841	0.4237	0.3682	0.027*	
C16	0.6493 (2)	0.32084 (15)	0.41608 (14)	0.0242 (5)	
H16	0.6834	0.3326	0.4672	0.029*	
C17	0.5975 (2)	0.24962 (14)	0.39862 (14)	0.0217 (5)	
H17	0.5962	0.2115	0.4376	0.026*	
C18	0.5477 (2)	0.23490 (13)	0.32337 (13)	0.0176 (5)	
C19	0.4872 (2)	0.16192 (13)	0.30408 (13)	0.0183 (5)	
H19	0.4852	0.1224	0.3416	0.022*	
C20	0.3255 (2)	0.07922 (13)	0.14288 (13)	0.0177 (5)	
C21	0.2646 (2)	0.00601 (13)	0.11886 (13)	0.0171 (5)	
C22	0.2388 (2)	−0.05078 (13)	0.17076 (14)	0.0201 (5)	
H22	0.2577	−0.0428	0.2247	0.024*	
C23	0.1856 (2)	−0.11876 (14)	0.14364 (15)	0.0238 (5)	
H23	0.1677	−0.1574	0.1791	0.029*	
C24	0.1585 (2)	−0.13070 (15)	0.06519 (15)	0.0280 (6)	
H24	0.1226	−0.1777	0.0468	0.034*	
C25	0.1834 (2)	−0.07436 (15)	0.01314 (15)	0.0306 (6)	
H25	0.1643	−0.0825	−0.0408	0.037*	
C26	0.2360 (2)	−0.00630 (14)	0.04003 (14)	0.0251 (5)	
H26	0.2528	0.0324	0.0044	0.030*	
N4	0.54975 (16)	0.28668 (11)	0.26515 (11)	0.0169 (4)	
N5	0.43675 (16)	0.15311 (11)	0.23469 (11)	0.0166 (4)	
N6	0.37943 (17)	0.08470 (11)	0.21587 (11)	0.0182 (4)	
H6	0.3781	0.0469	0.2494	0.022*	
O2	0.32882 (14)	0.13387 (9)	0.09676 (9)	0.0185 (3)	
N7	0.60837 (18)	0.15065 (12)	0.13028 (12)	0.0241 (5)	
C27	0.6953 (2)	0.12439 (14)	0.16274 (14)	0.0205 (5)	
S1	0.82003 (6)	0.09015 (4)	0.20678 (4)	0.03524 (17)	
N8	0.19349 (18)	0.28742 (12)	0.14346 (12)	0.0224 (4)	
O3	0.27176 (15)	0.26897 (10)	0.19789 (9)	0.0215 (4)	
O4	0.22281 (15)	0.28812 (10)	0.07601 (9)	0.0231 (4)	
O5	0.09194 (16)	0.30532 (14)	0.15714 (13)	0.0452 (6)	
N9	0.23513 (19)	0.50814 (12)	−0.13305 (12)	0.0253 (5)	
O6	0.34435 (15)	0.52196 (10)	−0.11161 (10)	0.0233 (4)	
O7	0.19053 (16)	0.44694 (11)	−0.11330 (11)	0.0348 (5)	
O8	0.17479 (18)	0.55663 (12)	−0.17225 (13)	0.0476 (6)	
O9	0.63720 (14)	0.31663 (9)	0.10280 (9)	0.0221 (4)	
H1W	0.7079	0.2927	0.1030	0.026*	
H2W	0.6549	0.3671	0.1067	0.026*	
O10	−0.06785 (18)	0.51994 (11)	−0.16636 (11)	0.0367 (5)	
H3W	−0.0834	0.5758	−0.1601	0.044*	
H4W	0.0136	0.5302	−0.1521	0.044*	
O11	0.85954 (17)	0.25203 (11)	0.09708 (13)	0.0391 (5)	
H5W	0.9348	0.2746	0.1091	0.047*	
H6W	0.8705	0.2113	0.1315	0.047*	
O12	−0.0686 (6)	0.5805 (4)	−0.0217 (4)	0.048 (2)*	0.328 (7)
H7W	−0.1083	0.5713	0.0226	0.057*	0.328 (7)
H8W	−0.0964	0.6204	−0.0517	0.057*	0.328 (7)

### (II) Aqua(nitrato-κ2O,O')[N'-(pyridin-2-ylmethylidene-κN)benzohydrazide-κ2N',O](thiocyanato-κN)neodymium(III) nitrate 2.33-hydrate Atomic displacement parameters (Å^2^)

**Table:**

	*U*^11^	*U*^22^	*U*^33^	*U*^12^	*U*^13^	*U*^23^
Nd1	0.01406 (6)	0.01405 (7)	0.01184 (6)	−0.00045 (5)	0.00001 (4)	0.00071 (5)
C1	0.0212 (12)	0.0197 (12)	0.0262 (13)	0.0032 (10)	0.0029 (10)	−0.0030 (10)
C2	0.0256 (13)	0.0228 (13)	0.0306 (14)	0.0014 (10)	0.0051 (11)	−0.0083 (11)
C3	0.0309 (14)	0.0337 (15)	0.0203 (13)	−0.0041 (11)	0.0070 (11)	−0.0114 (11)
C4	0.0313 (14)	0.0292 (13)	0.0166 (12)	−0.0056 (11)	0.0038 (10)	−0.0014 (10)
C5	0.0154 (10)	0.0212 (12)	0.0174 (11)	−0.0031 (9)	0.0018 (9)	−0.0002 (9)
C6	0.0203 (12)	0.0193 (12)	0.0160 (11)	−0.0030 (9)	0.0008 (9)	0.0026 (9)
C7	0.0145 (11)	0.0183 (11)	0.0167 (11)	−0.0032 (9)	0.0015 (9)	−0.0003 (9)
C8	0.0195 (11)	0.0175 (11)	0.0201 (12)	−0.0011 (9)	0.0064 (9)	0.0007 (9)
C9	0.0415 (16)	0.0279 (14)	0.0183 (12)	0.0108 (12)	0.0047 (11)	0.0009 (10)
C10	0.0474 (17)	0.0265 (14)	0.0297 (15)	0.0148 (13)	0.0135 (13)	0.0068 (12)
C11	0.0336 (14)	0.0175 (12)	0.0377 (15)	0.0028 (11)	0.0194 (12)	−0.0021 (11)
C12	0.0278 (14)	0.0256 (13)	0.0288 (14)	−0.0054 (11)	0.0073 (11)	−0.0086 (11)
C13	0.0195 (12)	0.0206 (12)	0.0224 (12)	−0.0019 (9)	0.0035 (10)	−0.0011 (10)
N1	0.0169 (9)	0.0197 (10)	0.0176 (10)	−0.0004 (8)	0.0026 (8)	−0.0006 (8)
N2	0.0183 (9)	0.0148 (9)	0.0164 (9)	0.0000 (8)	0.0015 (8)	0.0003 (8)
N3	0.0249 (10)	0.0142 (9)	0.0154 (9)	0.0010 (8)	0.0017 (8)	0.0016 (8)
O1	0.0220 (8)	0.0162 (8)	0.0146 (8)	−0.0004 (7)	0.0004 (7)	0.0008 (6)
C14	0.0186 (11)	0.0219 (12)	0.0187 (12)	−0.0030 (9)	−0.0013 (9)	0.0012 (10)
C15	0.0199 (12)	0.0233 (13)	0.0231 (13)	−0.0022 (10)	−0.0031 (10)	−0.0024 (10)
C16	0.0225 (12)	0.0319 (14)	0.0165 (12)	0.0001 (11)	−0.0043 (10)	−0.0027 (10)
C17	0.0224 (12)	0.0256 (13)	0.0162 (11)	0.0018 (10)	−0.0014 (9)	0.0028 (10)
C18	0.0167 (11)	0.0191 (12)	0.0167 (11)	0.0025 (9)	0.0011 (9)	0.0005 (9)
C19	0.0191 (11)	0.0181 (12)	0.0172 (11)	0.0013 (9)	−0.0001 (9)	0.0033 (9)
C20	0.0167 (11)	0.0169 (11)	0.0195 (11)	0.0023 (9)	0.0020 (9)	0.0000 (9)
C21	0.0175 (11)	0.0151 (11)	0.0192 (12)	0.0000 (9)	0.0036 (9)	−0.0016 (9)
C22	0.0217 (12)	0.0205 (12)	0.0183 (12)	−0.0006 (10)	0.0033 (10)	0.0008 (9)
C23	0.0250 (13)	0.0191 (12)	0.0282 (13)	−0.0024 (10)	0.0064 (11)	0.0031 (10)
C24	0.0311 (14)	0.0206 (13)	0.0330 (15)	−0.0074 (11)	0.0061 (12)	−0.0061 (11)
C25	0.0400 (16)	0.0287 (14)	0.0222 (13)	−0.0096 (12)	−0.0008 (12)	−0.0030 (11)
C26	0.0325 (14)	0.0216 (13)	0.0211 (13)	−0.0045 (11)	0.0025 (11)	0.0025 (10)
N4	0.0159 (9)	0.0196 (10)	0.0148 (9)	−0.0006 (8)	0.0005 (8)	−0.0006 (8)
N5	0.0153 (9)	0.0159 (9)	0.0183 (10)	−0.0002 (7)	0.0001 (8)	−0.0002 (8)
N6	0.0213 (10)	0.0143 (9)	0.0184 (10)	−0.0018 (8)	−0.0001 (8)	0.0039 (8)
O2	0.0222 (8)	0.0169 (8)	0.0156 (8)	−0.0018 (7)	−0.0009 (7)	0.0019 (6)
N7	0.0209 (11)	0.0265 (11)	0.0250 (11)	0.0033 (9)	0.0027 (9)	−0.0005 (9)
C27	0.0247 (13)	0.0194 (12)	0.0183 (12)	0.0004 (10)	0.0059 (10)	−0.0025 (9)
S1	0.0328 (4)	0.0426 (4)	0.0277 (4)	0.0155 (3)	−0.0071 (3)	0.0002 (3)
N8	0.0184 (10)	0.0214 (11)	0.0269 (11)	0.0003 (8)	0.0011 (9)	−0.0031 (9)
O3	0.0208 (8)	0.0259 (9)	0.0174 (8)	0.0010 (7)	0.0008 (7)	0.0026 (7)
O4	0.0253 (9)	0.0251 (9)	0.0180 (8)	−0.0002 (7)	−0.0014 (7)	0.0013 (7)
O5	0.0179 (10)	0.0695 (16)	0.0482 (13)	0.0084 (10)	0.0046 (9)	−0.0150 (11)
N9	0.0264 (11)	0.0280 (12)	0.0201 (11)	−0.0027 (9)	−0.0031 (9)	0.0032 (9)
O6	0.0213 (9)	0.0255 (9)	0.0224 (9)	−0.0044 (7)	−0.0006 (7)	0.0032 (7)
O7	0.0306 (10)	0.0330 (11)	0.0393 (11)	−0.0134 (8)	−0.0022 (9)	0.0082 (9)
O8	0.0330 (11)	0.0454 (13)	0.0599 (15)	−0.0010 (10)	−0.0136 (10)	0.0258 (11)
O9	0.0184 (8)	0.0200 (9)	0.0280 (9)	−0.0016 (7)	0.0036 (7)	0.0013 (7)
O10	0.0356 (11)	0.0296 (10)	0.0425 (12)	0.0019 (9)	−0.0059 (9)	−0.0125 (9)
O11	0.0233 (10)	0.0345 (11)	0.0588 (14)	−0.0023 (8)	0.0013 (9)	−0.0024 (10)

### (II) Aqua(nitrato-κ2O,O')[N'-(pyridin-2-ylmethylidene-κN)benzohydrazide-κ2N',O](thiocyanato-κN)neodymium(III) nitrate 2.33-hydrate Geometric parameters (Å, º)

**Table:**

Nd1—O9	2.4459 (16)	C15—C16	1.376 (3)
Nd1—O2	2.4796 (15)	C15—H15	0.9500
Nd1—O1	2.5063 (15)	C16—C17	1.387 (3)
Nd1—N7	2.512 (2)	C16—H16	0.9500
Nd1—O3	2.5568 (17)	C17—C18	1.388 (3)
Nd1—N5	2.6479 (19)	C17—H17	0.9500
Nd1—N2	2.6491 (18)	C18—N4	1.358 (3)
Nd1—O4	2.6558 (17)	C18—C19	1.461 (3)
Nd1—N4	2.6985 (18)	C19—N5	1.283 (3)
Nd1—N1	2.7051 (19)	C19—H19	0.9500
C1—N1	1.344 (3)	C20—O2	1.248 (3)
C1—C2	1.383 (3)	C20—N6	1.347 (3)
C1—H1	0.9500	C20—C21	1.482 (3)
C2—C3	1.378 (4)	C21—C26	1.390 (3)
C2—H2	0.9500	C21—C22	1.393 (3)
C3—C4	1.382 (4)	C22—C23	1.383 (3)
C3—H3A	0.9500	C22—H22	0.9500
C4—C5	1.383 (3)	C23—C24	1.381 (4)
C4—H4	0.9500	C23—H23	0.9500
C5—N1	1.352 (3)	C24—C25	1.386 (4)
C5—C6	1.458 (3)	C24—H24	0.9500
C6—N2	1.286 (3)	C25—C26	1.380 (3)
C6—H6A	0.9500	C25—H25	0.9500
C7—O1	1.246 (3)	C26—H26	0.9500
C7—N3	1.348 (3)	N5—N6	1.374 (3)
C7—C8	1.480 (3)	N6—H6	0.8800
C8—C13	1.390 (3)	N7—C27	1.166 (3)
C8—C9	1.396 (3)	C27—S1	1.633 (3)
C9—C10	1.385 (4)	N8—O5	1.237 (3)
C9—H9	0.9500	N8—O3	1.258 (3)
C10—C11	1.379 (4)	N8—O4	1.259 (3)
C10—H10	0.9500	N9—O8	1.237 (3)
C11—C12	1.381 (4)	N9—O7	1.242 (3)
C11—H11	0.9500	N9—O6	1.267 (3)
C12—C13	1.385 (3)	O9—H1W	0.8997
C12—H12	0.9500	O9—H2W	0.9000
C13—H13	0.9500	O10—H3W	0.9942
N2—N3	1.374 (3)	O10—H4W	0.9400
N3—H3	0.8800	O11—H5W	0.9362
C14—N4	1.337 (3)	O11—H6W	0.9263
C14—C15	1.388 (3)	O12—H7W	0.9498
C14—H14	0.9500	O12—H8W	0.9021
			
O9—Nd1—O2	145.38 (5)	C13—C12—H12	119.7
O9—Nd1—O1	74.54 (5)	C12—C13—C8	120.0 (2)
O2—Nd1—O1	138.15 (5)	C12—C13—H13	120.0
O9—Nd1—N7	72.97 (6)	C8—C13—H13	120.0
O2—Nd1—N7	78.31 (6)	C1—N1—C5	117.1 (2)
O1—Nd1—N7	142.53 (6)	C1—N1—Nd1	121.75 (16)
O9—Nd1—O3	139.59 (5)	C5—N1—Nd1	120.94 (15)
O2—Nd1—O3	74.53 (5)	C6—N2—N3	117.68 (19)
O1—Nd1—O3	69.76 (5)	C6—N2—Nd1	125.03 (15)
N7—Nd1—O3	129.95 (6)	N3—N2—Nd1	117.27 (13)
O9—Nd1—N5	121.20 (6)	C7—N3—N2	116.14 (18)
O2—Nd1—N5	60.43 (5)	C7—N3—H3	121.9
O1—Nd1—N5	118.47 (5)	N2—N3—H3	121.9
N7—Nd1—N5	65.93 (6)	C7—O1—Nd1	125.22 (14)
O3—Nd1—N5	64.16 (5)	N4—C14—C15	123.6 (2)
O9—Nd1—N2	70.60 (6)	N4—C14—H14	118.2
O2—Nd1—N2	111.58 (5)	C15—C14—H14	118.2
O1—Nd1—N2	60.42 (5)	C16—C15—C14	119.1 (2)
N7—Nd1—N2	123.01 (6)	C16—C15—H15	120.4
O3—Nd1—N2	105.96 (5)	C14—C15—H15	120.4
N5—Nd1—N2	168.03 (6)	C15—C16—C17	118.6 (2)
O9—Nd1—O4	132.39 (5)	C15—C16—H16	120.7
O2—Nd1—O4	69.76 (5)	C17—C16—H16	120.7
O1—Nd1—O4	70.50 (5)	C16—C17—C18	119.0 (2)
N7—Nd1—O4	146.92 (6)	C16—C17—H17	120.5
O3—Nd1—O4	48.83 (5)	C18—C17—H17	120.5
N5—Nd1—O4	103.70 (5)	N4—C18—C17	122.9 (2)
N2—Nd1—O4	64.41 (5)	N4—C18—C19	116.9 (2)
O9—Nd1—N4	75.29 (6)	C17—C18—C19	120.2 (2)
O2—Nd1—N4	120.12 (5)	N5—C19—C18	117.6 (2)
O1—Nd1—N4	71.15 (5)	N5—C19—H19	121.2
N7—Nd1—N4	82.92 (6)	C18—C19—H19	121.2
O3—Nd1—N4	75.78 (5)	O2—C20—N6	120.7 (2)
N5—Nd1—N4	59.94 (6)	O2—C20—C21	121.3 (2)
N2—Nd1—N4	126.14 (6)	N6—C20—C21	118.0 (2)
O4—Nd1—N4	120.40 (5)	C26—C21—C22	119.4 (2)
O9—Nd1—N1	84.45 (6)	C26—C21—C20	117.2 (2)
O2—Nd1—N1	69.48 (5)	C22—C21—C20	123.4 (2)
O1—Nd1—N1	119.99 (5)	C23—C22—C21	119.9 (2)
N7—Nd1—N1	74.73 (6)	C23—C22—H22	120.0
O3—Nd1—N1	129.94 (5)	C21—C22—H22	120.0
N5—Nd1—N1	120.52 (6)	C24—C23—C22	120.2 (2)
N2—Nd1—N1	59.61 (6)	C24—C23—H23	119.9
O4—Nd1—N1	85.97 (5)	C22—C23—H23	119.9
N4—Nd1—N1	153.44 (6)	C23—C24—C25	120.2 (2)
N1—C1—C2	123.0 (2)	C23—C24—H24	119.9
N1—C1—H1	118.5	C25—C24—H24	119.9
C2—C1—H1	118.5	C26—C25—C24	119.7 (2)
C3—C2—C1	119.3 (2)	C26—C25—H25	120.2
C3—C2—H2	120.4	C24—C25—H25	120.2
C1—C2—H2	120.4	C25—C26—C21	120.5 (2)
C2—C3—C4	118.6 (2)	C25—C26—H26	119.7
C2—C3—H3A	120.7	C21—C26—H26	119.7
C4—C3—H3A	120.7	C14—N4—C18	116.8 (2)
C3—C4—C5	119.1 (2)	C14—N4—Nd1	122.63 (15)
C3—C4—H4	120.5	C18—N4—Nd1	120.55 (14)
C5—C4—H4	120.5	C19—N5—N6	118.13 (19)
N1—C5—C4	122.9 (2)	C19—N5—Nd1	124.82 (15)
N1—C5—C6	116.6 (2)	N6—N5—Nd1	116.85 (13)
C4—C5—C6	120.5 (2)	C20—N6—N5	115.70 (18)
N2—C6—C5	117.6 (2)	C20—N6—H6	122.2
N2—C6—H6A	121.2	N5—N6—H6	122.2
C5—C6—H6A	121.2	C20—O2—Nd1	125.59 (14)
O1—C7—N3	120.7 (2)	C27—N7—Nd1	149.40 (19)
O1—C7—C8	121.7 (2)	N7—C27—S1	177.8 (2)
N3—C7—C8	117.6 (2)	O5—N8—O3	120.1 (2)
C13—C8—C9	119.2 (2)	O5—N8—O4	122.0 (2)
C13—C8—C7	118.5 (2)	O3—N8—O4	117.91 (19)
C9—C8—C7	122.3 (2)	N8—O3—Nd1	98.98 (13)
C10—C9—C8	120.1 (2)	N8—O4—Nd1	94.19 (12)
C10—C9—H9	119.9	O8—N9—O7	121.5 (2)
C8—C9—H9	119.9	O8—N9—O6	119.1 (2)
C11—C10—C9	120.3 (3)	O7—N9—O6	119.5 (2)
C11—C10—H10	119.8	Nd1—O9—H1W	124.7
C9—C10—H10	119.8	Nd1—O9—H2W	128.4
C10—C11—C12	119.7 (2)	H1W—O9—H2W	105.2
C10—C11—H11	120.1	H3W—O10—H4W	88.1
C12—C11—H11	120.1	H5W—O11—H6W	97.3
C11—C12—C13	120.6 (2)	H7W—O12—H8W	115.9
C11—C12—H12	119.7		
			
N1—C1—C2—C3	1.1 (4)	C15—C16—C17—C18	−0.6 (4)
C1—C2—C3—C4	−0.7 (4)	C16—C17—C18—N4	1.6 (4)
C2—C3—C4—C5	−0.1 (4)	C16—C17—C18—C19	−176.9 (2)
C3—C4—C5—N1	0.5 (4)	N4—C18—C19—N5	−2.5 (3)
C3—C4—C5—C6	−179.1 (2)	C17—C18—C19—N5	176.0 (2)
N1—C5—C6—N2	−2.1 (3)	O2—C20—C21—C26	14.5 (3)
C4—C5—C6—N2	177.6 (2)	N6—C20—C21—C26	−164.2 (2)
O1—C7—C8—C13	22.9 (3)	O2—C20—C21—C22	−167.5 (2)
N3—C7—C8—C13	−156.3 (2)	N6—C20—C21—C22	13.8 (3)
O1—C7—C8—C9	−156.0 (2)	C26—C21—C22—C23	0.3 (4)
N3—C7—C8—C9	24.8 (3)	C20—C21—C22—C23	−177.7 (2)
C13—C8—C9—C10	1.6 (4)	C21—C22—C23—C24	0.3 (4)
C7—C8—C9—C10	−179.4 (2)	C22—C23—C24—C25	−0.6 (4)
C8—C9—C10—C11	−1.3 (4)	C23—C24—C25—C26	0.3 (4)
C9—C10—C11—C12	0.0 (4)	C24—C25—C26—C21	0.2 (4)
C10—C11—C12—C13	0.9 (4)	C22—C21—C26—C25	−0.5 (4)
C11—C12—C13—C8	−0.5 (4)	C20—C21—C26—C25	177.5 (2)
C9—C8—C13—C12	−0.8 (4)	C15—C14—N4—C18	−1.1 (3)
C7—C8—C13—C12	−179.7 (2)	C15—C14—N4—Nd1	177.88 (18)
C2—C1—N1—C5	−0.7 (3)	C17—C18—N4—C14	−0.7 (3)
C2—C1—N1—Nd1	174.43 (18)	C19—C18—N4—C14	177.8 (2)
C4—C5—N1—C1	−0.2 (3)	C17—C18—N4—Nd1	−179.75 (17)
C6—C5—N1—C1	179.5 (2)	C19—C18—N4—Nd1	−1.2 (3)
C4—C5—N1—Nd1	−175.31 (18)	C18—C19—N5—N6	−179.94 (19)
C6—C5—N1—Nd1	4.4 (3)	C18—C19—N5—Nd1	5.4 (3)
C5—C6—N2—N3	−179.59 (19)	O2—C20—N6—N5	−0.1 (3)
C5—C6—N2—Nd1	−1.3 (3)	C21—C20—N6—N5	178.61 (19)
O1—C7—N3—N2	4.2 (3)	C19—N5—N6—C20	178.4 (2)
C8—C7—N3—N2	−176.61 (19)	Nd1—N5—N6—C20	−6.5 (2)
C6—N2—N3—C7	177.8 (2)	N6—C20—O2—Nd1	7.8 (3)
Nd1—N2—N3—C7	−0.6 (2)	C21—C20—O2—Nd1	−170.89 (15)
N3—C7—O1—Nd1	−6.2 (3)	O5—N8—O3—Nd1	−175.8 (2)
C8—C7—O1—Nd1	174.64 (15)	O4—N8—O3—Nd1	3.1 (2)
N4—C14—C15—C16	2.1 (4)	O5—N8—O4—Nd1	175.9 (2)
C14—C15—C16—C17	−1.1 (4)	O3—N8—O4—Nd1	−2.9 (2)

### (II) Aqua(nitrato-κ2O,O')[N'-(pyridin-2-ylmethylidene-κN)benzohydrazide-κ2N',O](thiocyanato-κN)neodymium(III) nitrate 2.33-hydrate Hydrogen-bond geometry (Å, º)

**Table:**

*D*—H···*A*	*D*—H	H···*A*	*D*···*A*	*D*—H···*A*
N3—H3···O6	0.88	1.98	2.847 (2)	168
N6—H6···O10^i^	0.88	1.92	2.754 (3)	159
O9—H1*W*···O11	0.90	1.86	2.760 (3)	174
O9—H2*W*···O6^ii^	0.90	1.93	2.816 (2)	168
O10—H3*W*···O5^iii^	0.99	2.07	3.055 (3)	171
O10—H4*W*···O8	0.94	1.95	2.824 (3)	154
O11—H5*W*···O5^iv^	0.94	1.94	2.859 (3)	166
O11—H6*W*···S1	0.93	2.58	3.460 (2)	159
O12—H7*W*···O7^iii^	0.95	1.95	2.902 (7)	180
O12—H8*W*···O5^iii^	0.90	2.25	3.072 (7)	151
O12—H8*W*···O4^iii^	0.90	2.14	2.957 (7)	149

Symmetry codes: (i) *x*+1/2, −*y*+1/2, *z*+1/2; (ii) −*x*+1, −*y*+1, −*z*; (iii) −*x*, −*y*+1, −*z*; (iv) *x*+1, *y*, *z*.
